# Analyzing Co-expression Networks with Network Skeleton Extraction

**DOI:** 10.21203/rs.3.rs-8989183/v1

**Published:** 2026-03-25

**Authors:** Eliza Duvall, Rosemary Braun

**Affiliations:** 1 Department of Molecular Biosciences, Weinberg College of Arts and Sciences, Northwestern University, Evanston, IL 60208, USA; 2 Santa Fe Institute, Santa Fe, NM 87501, USA; 3 Engineering Sciences and Applied Mathematics, McCormick School of Engineering, Northwestern University, Evanston, IL 60208, USA; 4 Physics and Astronomy, Weinberg College of Arts and Sciences, Evanston, IL 60208, USA; 5 NSF-Simons National Institute for Theory and Mathematics in Biology, Chicago, IL 60611, USA

**Keywords:** co-expression, spectral sparsification, network biology

## Abstract

**Background:**

A central question in systems biology is to infer how genes work together to perform a specific function. While publicly available gene regulatory networks are commonly used to analyze experimental data, these networks may contain inaccuracies or inconsistencies across databases, especially for less well-studied organisms or systems where regulatory networks change over time. Data-driven network reconstruction from co-expression data is an appealing alternative, but requires sparsification to yield biologically representative networks. Threshold-based sparsification can lead to an excessively fragmented network, masking the relationship between genes and neglecting the “strength of weak ties”, wherein a critical but weak relationship may serve as a vital link between two subnetworks.

**Results:**

Here we present Network Skeleton Extraction (NSE), a co-expression network generation method using spectral sparsification to sparsify co-expression statistics into minimal co-expression graphs. Spectral sparsification has the advantage of maintaining connections among genes in a manner that preserves the coarse-grained structure of the input graph. In our method, the degree of sparsification is optimized by predicting each gene’s expression as a function of its connected genes at each sparsification level. This yields networks that are maximally sparse while still being predictive of gene expression. A probabilistic model also provides a null distribution of networks with similar spectral properties against which inferred networks can be compared. We illustrate the method by applying it to Xenopus transcriptome data from four cell types and six developmental stages to obtain networks specific to the organism, cell type, and developmental stage.

**Conclusions:**

By applying NSE to pre-defined gene sets in a phenotype-conditional manner, we can identify pathways whose coordination differs significantly across cell types and developmental stages.

## 1 Background

A central question in systems biology is to elucidate how genes work together to perform specific functions. Networks provide a natural analytical framework for analyzing these relationships by representing interactions between genes, rather than focusing on individual genes in isolation. Various types of biological networks, such as gene regulatory networks, protein–protein interaction networks, or gene co-expression networks, offer different approaches to decipher complex biological dynamics.

Here we focus on gene co-expression network generation and analysis. These data-derived networks provide an approach for capturing novel or context-specific relationships that may not appear in publicly-available curated databases [1, 2]. Generating condition–specific networks enables comparisons across conditions, such as cell types, exposures, or developmental stages, revealing differences at the network level [3]. For example, these analyses allow the identification of context–dependent gene associations [4, 5], the discovery of pathways that are activated or suppressed under different conditions, and the pinpointing of genes that serve as key regulators in specific biological states [6].

We define co-expression networks as graphs in which nodes represent genes, and edges capture pairwise statistical associations in gene expression, such as correlation or mutual information. Strongly associated pairs are assumed to follow the “guilt by association” principle, wherein genes with highly coordinated expression patterns are more likely to share functional roles [7]. When generating a co-expression network, edge weights are first calculated for all possible pairwise relationships, including those with near–zero correlations, which indicate little to no consistent co-expression between genes. Decisions must then be made regarding which edges of the complete graph to keep. Sparsifying the network by removing irrelevant edges simplifies the graph, reduces noise, clarifies biologically meaningful patterns, and makes downstream analyses more interpretable and efficient.

In general, the goal of network sparsification is to remove edges from a graph while preserving key structural properties [8]. Different strategies may be used depending on the biological question and the type of data. Sparsification via thresholding is the most common approach in co-expression studies, wherein edges with correlation magnitudes below a chosen cutoff are removed. While this method is intuitive and can be effective in emphasizing strong gene–gene associations, it can remove weak yet functionally important connections. Indeed, thresholded networks are likely to fragment into multiple subnetworks, obscuring the relationship between functional modules. Similarly, *k*-nearest neighbor sparsification (which retains the *k* strongest edges for each node) likewise does not guarantee a connected network, and additionally imposes an artificial degree distribution. Another method, ARACNe, first uses mutual information to sparsify networks via thresholding; then, for every set of three genes that are connected in a triangle, ARACNe removes the edge with the lowest value of mutual information, retaining only direct regulatory relationships inferred from the data [9].

While these approaches may be suitable for identifying networks based on guilt-by-association, thresholding fails to consider the “strength of weak ties,” wherein a critical but weak relationship connects two distinct sub-networks [10]. In biological systems, there may be loosely associated genes that play a critical role by connecting otherwise large, distinct modules and enabling coordination between biological processes. An excessively fragmented network may overlook the importance of multi-functional genes by associating each gene with a single module, despite the fact that some are known to be involved in multiple biological pathways. Careful sparsification is therefore essential to preserve key structural properties and maintain the integrity of the co-expression network in a mathematically rigorous way.

It is often of interest to compare the co-expression graphs pertaining to different biological conditions. Approaches to compare co-expression networks can be classified into three major groups, each operating at different scales [3]. The first focuses on global network features, such as modularity or overall network topology. For example, researchers may compare the overall connectivity between healthy and diseased networks to detect broad shifts in transcriptional coordination [11–13]. The second are module-based methods, which examine groups of co-regulated genes that have differential connectivity across conditions. Some methods identify modules corresponding to similar biological pathways and test whether they show altered connectivity in different conditions [11, 14, 15]. The third focuses on single–gene changes, investigating how the network neighborhood of individual genes shifts under different conditions, which can highlight key regulators or hub genes that mediate context-specific processes [4, 11, 16]. By organizing thousands of genes into structured networks, co-expression analysis provides a flexible framework for uncovering novel pathways [3, 5], predicting gene function [6, 14, 15], identifying disease-associated genes [6, 17], and generating testable hypotheses for experimental validation [3].

A widely used co-expression network generation and analysis method is Weighted Gene Co-Expression Network Analysis (WGCNA) [18]. WGCNA begins by constructing a complete graph in which edges are weighted by the correlations between all gene pairs. The network is then sparsified using a threshold to yield modules of highly correlated genes. Each module is then summarized by its eigengene, the first principal component of its gene expression profile, which captures the overall expression trend. Additionally, DiffCoEx is an extension of WCGNA that is designed to identify modules of genes whose co-expression relationships differ between conditions rather than modules that are simply co-expressed in both [19].

Other methods, such as Differential Co–Expression Gene Linkage (DCGL), focus on the gene level to identify differentially co-expressed links across conditions to infer potential regulatory changes [20]. After constructing a co-expression network for each condition, DCGL tests whether the correlation between gene pairs significantly changes between conditions and summarizes which genes have a large number of differentially co-expressed links. There are also methods that test the significance of differentially co-expressed networks by generating permutation–based null models [21, 22].

In all of these analyses, complete co-expression graphs for each condition are first sparsified before being compared. The results of the comparative analyses therefore depend on the sparsification approach used. In particular, methods that focus on differences in global network features, such as modularity or overall network topology, could be compromised by sparsification strategies that change these characteristics. We propose here that a strategy that preserves the networks’ spectral properties (and hence its global structural and dynamical characteristics [23, 24]) may provide a more faithful representation of the co-expression network than simple thresholding. Preservation of structural features enables comparative analysis across conditions to systematically identify meaningful connectivity differences between networks, revealing condition–specific alterations in network architecture and function.

Properties such as the network’s global connectivity and flow through the network can be quantified by the the spectral properties — the eigenvalues and eigenvectors — of the graph Laplacian [23, 24], a matrix that encodes the network’s edge weights and node degrees. It can be shown that the number of zero eigenvalues reflects how many disjoint components the graph contains, and the spectral gap (the difference between the first [zero] and second eigenvalue) quantitatively measures the network’s overall connectivity. As the spectral gap approaches zero, the graph becomes increasingly susceptible to fragmentation (it is more easily cut) and flow through the network is reduced. A sparsification method that can approximately preserve the eigenvalue spectrum of the graph Laplacian will retain the original network’s structural properties [8, 25], allowing the resulting network “skeleton” to approximate the original graph and serve as a proxy which mimics behaviors and characteristics.

Here we present Network Skeleton Extraction (NSE), a sparsification method that approximately preserves the spectral properties of the original complete graph. Our method adapts the spectral sparsification approach proposed by Spielman and Srivastava [8, 25], which was demonstrated to preserve spectral characteristics. The resulting skeletons may then be compared across conditions to reveal differences in network connectivity. By applying NSE to pre-defined gene sets (lists of genes known to be associated with particular biological functions), we reduce the method’s computational complexity while providing mechanistically interpretable insights into the underlying biological processes represented by each network. Network skeletons generated from different conditions (e.g., from data from different cell types or developmental stages) can then be compared to a null distribution of spectrally–matched random graphs to identify statistically significant changes in edges or overall connectivity. Below we describe the NSE method, the statistical testing framework, and demonstrate its utility .

## 2 Methods

### 2.1 The Network Skeleton Extraction (NSE) Method

#### 2.1.1 Generate Complete Network

**Figure 1:**
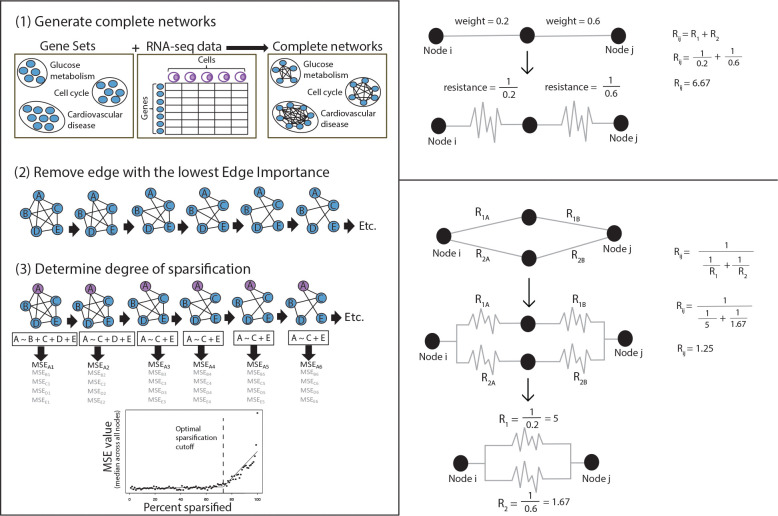
Left: Summary of selecting edges using spectral sparsification: (1) Complete networks are generated using experimental data (RNA-seq) to calculate correlations between every pair of genes within the gene set. (2) Edges with lowest $I_{ij}$ are iteratively removed, recalculating $I_{ij}$ at each iteration. (3) The optimal degree of sparsification is determined using the elbow in the median MSE of the regression model for each gene as a function of its neighbors. Right: Examples of calculating effective resistance between two nodes for serial and parallel paths.

NSE begins by constructing a complete co-expression network for a given gene set (e.g., genes involved in a common pathway or biological function). This requires two inputs: the list of genes in the gene set and transcriptomic data from which gene co-expression statistics can be calculated. Gene sets are defined as collections of genes associated with specific biological processes or cellular functions (e.g., glucose metabolism, TGF-beta signaling, or the cell cycle), Figure 1A; these may be user–defined or obtained from databases such as gene ontology [26], KEGG [1], or Reactome [2]. Leveraging gene sets as prior knowledge decreases the computational complexity of the network inference problem, reduces the probability of spurious links between unrelated genes, and facilitates interpretation of the results.

We then assign a weight $w_{ij}$ to every pair of genes (i,j) in the set as $w_{ij}=|Cor(i,j)|$, i.e., the absolute value of their correlation in the gene expression data. This approach assumes that strong co-expression, whether positive or negative, indicates related responses, following the “guilt by association” principle [7]. The correlation can be calculated using Pearson or Spearman (rank) correlation, where Spearman is recommended when fewer samples are available; other co-expression statistics (such as mutual information) may also be used [27]. The result is a complete (all-to-all) graph with edge weights that reflect the degree of co-expression between gene pairs.*

#### 2.1.2 Calculating Edge Importance

Before the network is sparsified, each edge is ranked according to an importance metric. Following prior work by Spielman and colleagues [8, 25], edge importance is measured by the strength of co-expression between gene pairs ($w_{ij}$) combined with the edge’s role in maintaining the overall network structure, measured by effective resistance. The effective resistance $R_{ij}$ can be understood by considering the network as an electrical circuit where each edge is a resistor with a resistance equal to $1/w_{ij}$. In this analogy, if a network has two nodes reachable only through a single edge, the resistance between them is equal to $1/w_{ij}$. However, when additional paths exist between two nodes, the effective resistance is reduced, similar to how resistance decreases in a parallel circuit. The effective resistance $R_{ij}$ thus quantifies the extent to which nodes i and j are (de)coupled given the topology of the whole network, and thus the flow between them via any path.

To calculate $R_{ij}$ from the weighted adjacency matrix $W=\left\{w_{ij}\right\}$, we compute the (unnormalized) graph Laplacian L=D-W, where D is the diagonal degree matrix such that $D_{ii}=\sum_{j}w_{ij}$. The effective resistance between nodes i and j is then calculated according to:

(1) $$R_{ij}={\left(e_{i}-e_{j}\right)}^{⊤}L^{+}\left(e_{i}-e_{j}\right),$$

where $L^{+}$ is the Moore–Penrose pseudoinverse of L and $e_{i},e_{j}$ denote vectors which are 1 at indices i or j respectively and 0 elsewhere. The edge importance $I_{ij}$ is defined as the product $I_{ij}=w_{ij}R_{ij}$.

Edges with both low $w_{ij}$ and $R_{ij}$ represent weak links with many alternative paths, and can likely be removed without compromising the network’s overall structure. By contrast, edges with a high $w_{ij}$ and $R_{ij}$ are more crucial to maintaining the network structure. $I_{ij}$ thus captures the significance of the direct path as well as the contribution of all alternative paths between the nodes, providing a comprehensive measure of an edge’s role in the network’s function. Table 1 describes the relationship between the contributions of $w_{ij}$ and $R_{ij}$. When $I_{ij}$ is zero, it indicates that there is no edge between the two nodes ($w_{ij}=0$). When $I_{ij}$ is very low, it suggests that both $w_{ij}$ is low and that there are many alternative paths between the nodes (low $R_{ij}$), implying that the direct edge is not crucial for maintaining connectivity. Conversely, when $I_{ij}$ is high, it indicates that either the edge weight $w_{ij}$ high, or that there are few alternative paths between the nodes (high $R_{ij}$), making the direct edge more essential for maintaining connectivity and network structure.

#### 2.1.3 Removing Edges with Least Importance

To sparsify the networks, Spielman and Srivastava [8] convert $I_{ij}$ into a probability by normalizing the edge importance across the graph $p_{ij}=I_{ij}/\sum_{ij}I_{ij}$ and sample a chosen fraction of the edges with replacement according to those probabilities, yielding a sparser graph. This sparsification method largely preserves the spectrum of the graph Laplacian L [8, 25, 28]. Preserving the spectrum of the original network is crucial because the eigenvalues reflect the network’s structural properties [24, 29], ensuring that aspects of the system’s behavior (including overall connectivity and flow through the network) is largely maintained. However, sampling edges from $p_{ij}$ selects a different set of edges every time it is conducted, resulting in nondeterministically sparsified networks.

Mercier et.al. applied this nondeterministic approach to sparsify a mobility network [30], a method particularly suited for such networks where edges are interchangeable (e.g., people are not always constrained to the same route and may substitute one street for another). Probabilistic sparsification is suitable when only the global characteristics of the network structure, and not the specific edges, are of interest. In contrast, it is desirable for biological networks to have interpretable interactions and reproducible results given the same conditions. We thus adopt a deterministic approach to ensure the selection of the same set of edges across repeated applications while still preserving the overall network structure.

In our deterministic sparsification approach, the network is sparsified by iteratively removing the edge with the lowest $I_{ij}$ (i.e., setting $w_{ij}$ to zero). The removal of an edge decreases the number of alternative paths between node pairs, leading to an increase in effective resistance across the network (Table 1); hence, following the removal of an edge, both $R_{ij}$ and $I_{ij}$ are recalculated for all edges before the next edge with the lowest non-zero $I_{ij}$ is selected for removal (Figure 1A). This greedy iterative process continues until all edges are considered.

While this method continuously ranks the edges from least to most important and therefore determines the order of their removal, it does not directly indicate the optimal extent of sparsification. We describe the stopping criterion in the following section.

#### 2.1.4 Determining the Degree of Sparsification

In NSE, we stop sparsifying the graph at the point where removing further edges compromises the ability to predict the expression of a gene as a function of its neighbors. Specifically, we fit a regression model to predict the expression level of each gene as a function of its connected genes at each stage of sparsification, as illustrated by Figure 1A. As the network becomes more sparse, the dependent gene (“A” in Figure 1A) will have fewer genes available to contribute to its expression prediction, impacting the model’s ability to accurately estimate gene expression and increasing its mean squared error (MSE).

NSE computes the MSE for all genes as a function of their neighbors the network is sparsified. When the median MSE across all genes in the network is plotted against the percentage of the network that has been sparsified, we find that the median MSE increases gradually with sparsification, until it exhibits an “elbow” at which the MSE increases sharply 1A. NSE uses the elbow as the optimal sparsification, beyond which the ability to predict the expression of a given gene based on its neighbors is compromised. This point is estimated with two intersecting lines that best fit to the data; the junction point where the curves meet is defined as the elbow [31] and sets the sparsification threshold.

To ensure that the skeleton remains connected, NSE limits sparsification to the point immediately before the network fragments into multiple components. The number of connected components in a network is given by the number of zero eigenvalues of the graph Laplacian [29]. NSE determines the sparsification level at which fragmentation first occurs, i.e., the point at which the zero eigenvalue has degeneracy two (or, equivalently, the point at which the spectral gap between the first and second eigenvalue is zero). It then fits the two intersecting lines to the MSE value *before* this point to find the optimal extent of sparsification. The resulting skeleton maintains a spectral gap greater than zero, ensuring that the network skeleton remains a single component.

### 2.2 Analyzing differences in network skeletons

When multiple biological conditions (e.g., developmental stages or cell types) are considered, separate network skeletons can be generated using the same underlying gene sets to enable direct comparison. Skeletons can be compared on a pairwise basis, or multiple skeletons may be evaluated against the same reference.

#### 2.2.1 Measuring network similarity

Computing similarities between network skeletons derived from different conditions can be approached in multiple ways, as different metrics capture distinct aspects of structural variation, including edge-level overlap, relative connectivity patterns, and global graph topology. Here we primarily use the normalized Hamming distance [32], as well as the Jaccard index [33] and spectral distance [23].

#### Normalized Hamming Distance

The normalized Hamming distance measures the fraction of edge positions that differ between two networks, capturing how much edge-level rewiring has occurred. In this implementation, adjacency matrices are first binarized such that edges with weight greater than zero are treated 1, and 0 otherwise. The normalized Hamming distance between two binarized network skeletons “A” and “B” is defined as

(2) $$D_{H}(A,B)=\frac{\sum_{i<j}\left|A_{ij}\;-\;B_{ij}\right|}{N(N\;-\;1)/2},$$

where $A_{ij}$ and $B_{ij}$ denote the entries of the *binarized* adjacency matrices corresponding to skeletons A and B, and N is the number of nodes (genes). This metric quantifies the proportion of edge presence/absence mismatches. A value of 0 indicates that the skeletons are identical, whereas larger values up to 1 reflect increasing levels of edge–level rewiring.

#### Jaccard Index

The Jaccard index measures how much two network skeletons overlap by quantifying the proportion of shared edges relative to all edges present in either network. This similarity metric provides an intuitive assessment of how similar the retained interaction sets are. Jaccard indices between network skeletons A and B are evaluated by

(3) $$S\left(A,B\right)=\frac{\left|{ℰ}_{A}\;\cap \;{ℰ}_{B}\right|}{\left|{ℰ}_{A}\;\cup \;{ℰ}_{B}\right|},$$

where ${ℰ}_{A}$ is the edge set of skeleton A, and likewise for B. The index equals 1 when there is no difference between two networks, and 0 when no edges are in common.

A drawback of the Jaccard index is that it is extremely sensitive to the sparsity of the graphs. In cases where both graphs are sparse, the denominator (edge set union) will be small, and a slight change in the numerator (edge set intersection) can make a large difference in Eq. 3. By contrast, the (normalized) Hamming distance (Eq. 2) is insensitive to the average sparsification level of the graphs being compared.

#### Spectral distance

The spectral distance compares networks based on their eigenvalue spectra derived from the graph Laplacian, which summarizes global connectivity patterns. In this implementation, similarity is assessed using the difference between the first (zero) and second eigenvalue of the Laplacian matrix, otherwise known as the Fiedler value or the algebraic connectivity. As noted, this value becomes smaller as the graph becomes sparser and less connected; when a graph is fragmented into two or more subgraphs, this value will be zero. The spectral distance between two skeletons thus measures how the *overall* connectivity of two graphs A and B differ:

(4) $$D_{spec}(A,B)=\left|\left(\lambda_{2}^{\left(A\right)}-\lambda_{1}^{\left(A\right)})-(\lambda_{2}^{\left(B\right)}-\lambda_{1}^{\left(B\right)}\right)\right|$$

(5) $$=\left|\lambda_{2}^{\left(A\right)}-\lambda_{2}^{\left(B\right)}\right|,$$

where $\lambda_{i}$ denotes the i-th eigenvalue of the graph Laplacian, sorted from smallest to largest, and hence $\lambda_{1}=0$. This metric quantifies differences in algebraic connectivity, capturing global structural changes in overall network cohesion rather than individual edge differences. A value of 0 indicates identical connectivity structure with respect to this spectral property, whereas larger values reflect increasing difference in network cohesiveness. (Note that as defined here, $D_{\text{spec}}(A,B)$ is bounded from above by $\frac{N}{\left(N-1\right)}max\left(\underset{i\in {𝒱}_{A}}{min}\left(d_{i}^{\left(A\right)}\right),\underset{j\in {𝒱}_{B}}{min}\left(d_{j}^{\left(B\right)}\right)\right)$, where $d_{i}^{\left(A\right)}$ denotes the node degrees for NSE A, and thus the max denotes the maximum minimal–node–degree of the two graphs. This bound increases with network size. Alternatively, the use of the normalized Laplacian would confine this on [0, 2], independent of network size.)

#### 2.2.2 Assessing statistical significance of similarities/differences

It is reasonable to ask how the amount of similarity between two condition–specific skeletons compares to what would be obtained from a random graph *with the same sparsity and spectral properties*. Spectrally–matched random graphs were created to enable a condition–specific similarity comparison. The procedure used to generate these spectrally matched random graphs and construct corresponding null distributions is outlined below.

Consider two network skeletons obtained from data of “Condition A” and “Condition B”. The data are randomly resampled without replacement generate an equal number of replicates as originally observed, effectively permuting the condition labels. The correlations, and hence the edge weights for the complete graphs, were recalculated from the resampled data. To prevent differentially expressed genes from inducing spurious correlations in the resampled data, the expression levels for each gene are first mean-centered across samples within each condition, thereby normalizing baseline expression levels prior to resampling and calculation of the edge weights.

From these resampled edge–weights, spectrally-matched random graphs were created by using the probabilistic sparsification described in [8], in which edges are kept with a probability according to their edge importance, $p_{ij}=I_{ij}/\sum_{i<j}I_{ij}$. Edges were sampled with this probability until the network density was equal to the original condition–specific observation, producing a random graph with the same sparsification level as the observed data and the approximate spectral properties of the resampled data. (We used the probabilistic method rather than our deterministic, iterative, MSE–based method here to improve computational efficiency and to introduce additional variability into the null model.)

Repeatedly doing this enabled us to generate an ensemble of network skeletons that differ from the reference skeleton in terms of which edges are kept, but still maintain the skeleton’s sparsification level. From this ensemble, we can compute a distribution of similarity metrics between the spectrally–matched random graphs. By comparing the similarity metrics between the observed “Condition A” and “Condition B” to the null distribution of similarity scores among spectrally–matched random graphs, we can assess whether “Condition A” and “Condition B” differ more or less than expected by chance.

## 3 Results and Discussion

### 3.1 Application to Xenopus development RNA-seq data

As a proof of concept, we applied our method to a transcriptome dataset from developing Xenopus embryos [34]. Xenopus is a valuable model organism in developmental biology due to its well–defined developmental stages and the ease with which gene expression can be manipulated to produce distinct cell lineages. Past research has demonstrated the epidermal and neural cell types originate from ectodermal tissue, whereas the mesodermal and endodermal cell types derive from mesendodermal tissue. At earlier stages of development, before cellular differentiation and lineage specification, cell types exhibit greater similarity to one another. Here we apply our method to RNA-seq data from Xenopus to study lineage– and stage–specific networks.

As previously described [34], ectodermal cells from early-stage Xenopus embryos were isolated and directed to differentiate into four distinct cell types (endoderm, mesoderm, epiderm, and neural) and six developmental stages (stage 9, stage 10, stage 10.5, stage 11, stage 12, and stage 13). These were then subject to bulk RNA sequencing, providing gene expression data across all lineages and stages (Table S-1, [34]). Edge weights were then assigned according the Pearson correlation of expression between all gene pairs in each gene set. The gene set data used for network construction were sourced from the publicly available KEGG database [1]; a total of 146 gene sets were considered, with sizes ranging from 7 to 192 nodes.

#### 3.1.1 Network Exploration

**Figure 2:**
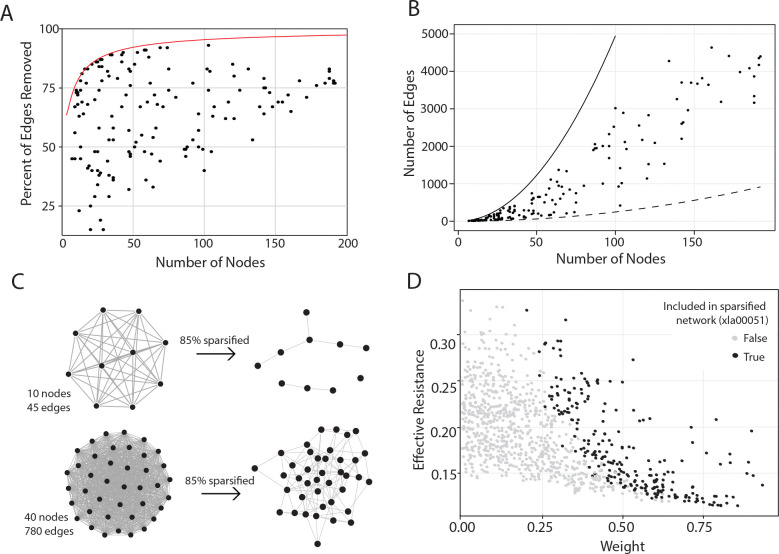
(A) The percent of edges removed as a function of the gene set size. The red line depicts 1-log(N)/N, the Erdős–Rényi (E-R) connectivity threshold. (B) The number of edges retained in the sparsified graph versus the number of nodes; the upper limit is the maximum possible edges (N(N-1)/2) and the lower curve illustrates 5% of the possible edges. (C) Two complete networks with 10 nodes (top) and 40 nodes (bottom), each sparsified to remove 85% of edges, in which the smaller network fragments into three components and the larger network remains in a single component. (Note that 85% is above the E-R threshold of 1-logN/N for a network of 10 nodes, but not for one of 40.) (D) Effective resistance ($R_{ij}$) vs. edge weight ($w_{ij}$) for an example network (xla00051) with 45 nodes and 990 possible edges; this network was sparsified to remove 77% of the initial edges (gray circles).

Here we explore the results of network skeletons generated using 146 gene sets and transcriptome data from all Xenopus samples (4 different cell types at 6 developmental time points). Details of the networks are given in Supplementary Information. Figure 2A shows how the percent of edges removed from the networks varies with respect to network size. Figure 2B shows the number of remaining edges relative to the number of nodes, with lines indicating the upper bound of a complete graph with no sparsification (N(N-1)/2 edges), and the bottom line indicating 95% sparsification (0.05 N(N-1)/2 edges). We see in Figure 2A that as network size increases, the range of edge sparsification narrows, indicating that larger networks can be more aggressively sparsified, whereas smaller networks are often intolerant to strong sparsification. This can be attributed both to the susceptibility of small networks to fragmentation, as well as the fact that in smaller networks the MSE is likely to increase faster (due to fewer predictors remaining as sparsification increases). Indeed, there are limits to the amount that the graph can be sparsified without fragmenting the network; the well-known connectivity threshold result from graph theory states that a Erdős–Rényi (E-R) random graph is almost surely disconnected when the fraction of edges drops below logN/N [35] (the red line in Figure 2), demonstrating that larger graphs are more tolerant of higher degrees of sparsification. By way of illustration, Figure 2C provides an example of a smaller network with 10 nodes and a larger network with 45 nodes both sparsified to remove 85% of edges. The smaller network fragments into multiple components, whereas the larger network is more robust to a higher degree of sparsification and remains in a single component.

In Figure 2D, we show the statistics for the edges kept/removed for a randomly selected mid-size Xenopus network (xla00051). This pathway has 45 genes and thus a maximum of 990 possible edges, but was sparsified to 23% of its edges (removing 77%, shown as gray circles). It can be seen that edges with high weights and any level of effective resistance remain in the final network, whereas extremely low-weight edges were removed regardless of $R_{ij}$. Discrimination based on $R_{ij}$ becomes apparent in the mid-weight edges. Notably, some edges with higher weights but lower $R_{ij}$ are removed, while others with lower weights (lower gene–gene correlation) are retained in the final network, in accordance with the “strength of weak ties” [10].

**Figure 3:**
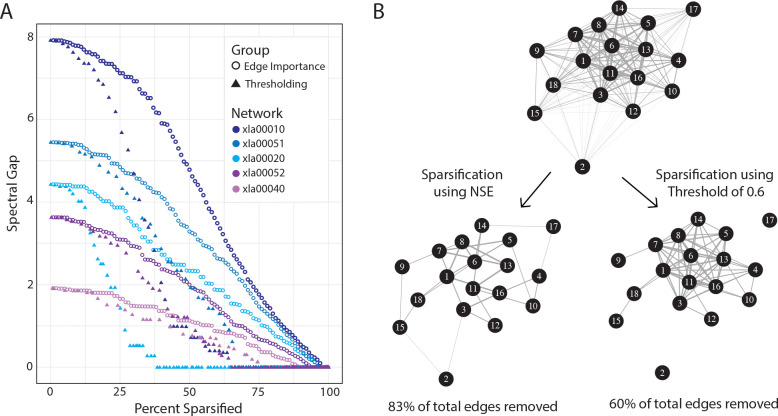
(A) Changes in the spectral gap as five networks are gradually sparsified, using thresholding or spectral sparsification. (B) A complete network (id: xla00515) with 18 nodes sparsified using NSE (left) and a correlation threshold of 0.6 (right). Edge thicknesses depict the correlation coefficient.

Recalling that our goal is to sparsify the network in a manner that best preserves its spectral properties, we now illustrate the change in the spectral gap as the networks are sparsified. Figure 3A compares the spectral gap for five networks using two sparsification methods: NSE and thresholding. For thresholding as a sparsification method, edges with absolute correlations below a threshold value were removed. We swept thresholds from 0 to 1 increasing by 0.01, and calculated the percent of edges removed and the resulting spectral gap for direct comparison with NSE. As expected, in both sparsification methods there is a decline in the spectral gap as the network becomes more sparse; however, this change is much more rapid by means of thresholding, and tends to reach 0 (indicating the splitting of the network into multiple disjoint subgraphs) at lower levels of sparsification than NSE. Figure 3B shows an example of a network sparsfied by removing 83% of edges using NSE, compared to the same network sparsified by removing 60% of edges using a threshold value of 0.6. While the network sparsified using NSE retained its spectral gap and remained in a single component, the network sparsified using a threshold fragmented, divorcing two genes completely from the whole network. The isolation of genes from the pathway compromises biological interpretability and can pose a problem for downstream analyses which seek to understand how specific genes impact the function of a pathway [3, 4, 11, 13, 16].

#### 3.1.2 Reproducibility and sensitivity to data subsampling

We next address the question of how reliable the NSE is, given that the input data is subject to random experimental sampling. We tested our expectations that we should see (i) similar networks when the input data differed at random, due to random sampling of replicates, and (ii) different sparsification outcomes when the underlying biology differs.

We studied the reproducibility with respect to random subsampling of replicates in two ways: In the “Group 1” tests, we randomly removed one replicate per each stage and cell type (effectively removing 24 of the 94 samples) and recalculated the sparsified network skeletons. In “Group 2”, we randomly selected 20% of the samples to generate network skeletons (i.e., excluding 80% of samples). The resulting networks were compared to a reference network generated using *all* samples. We quantified the similarity using the normalized Hamming distance (Eq 2), and other similarity metrics (Supplementary Figure S-1), to analyze how subsampling alters the retained edges. In each case, we repeated the subsampling eight times for all 146 pathways to generate a distribution of similarity scores.

**Figure 4:**
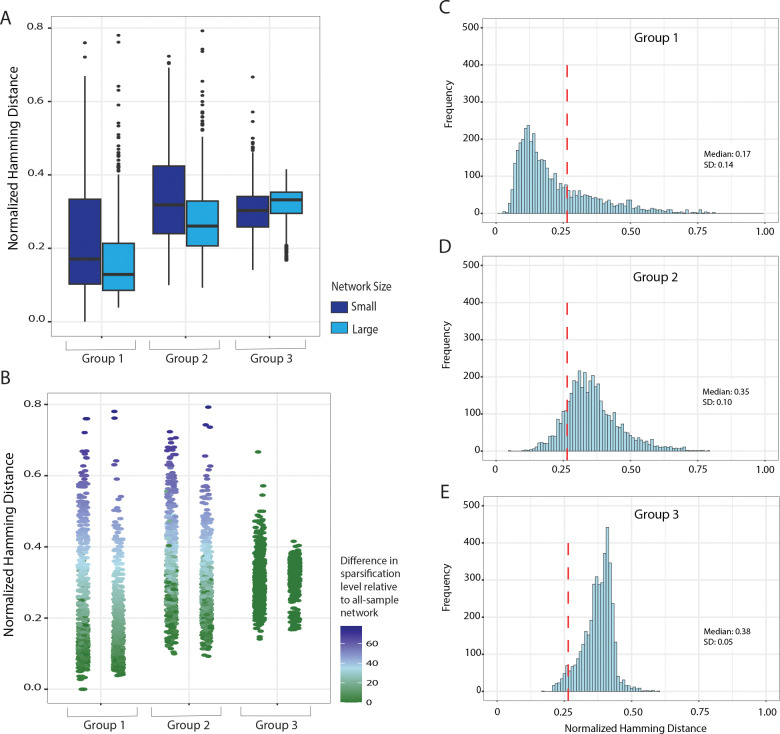
(A) Normalized Hamming distances (=0 if exactly the same) of three groups of networks when compared to the all–sample reference network. Group 1 are networks created from subsets of the data with one replicate per cell type per stage removed. Group 2 are networks created from subsets of the data with a random selection of 20% of the samples. Group 3 are probabilistically–sparsified networks (i.e., following the non-deterministic sparsification of Spielman & Srivastava) obtained by sparsifying to the same extent as the reference networks using the edge importance as a probability; Group 3 thus represents a “spectrally–matched null model”. Plots are split by network size where small < 50 nodes (n = 74) and large ≥ 50 nodes (n=76)(B) Results in (A) shown as a dot plot colored by the difference in sparsification threshold between the all–sample reference network and each group. Higher Hamming distance scores are associated with largely different sparsification levels compared to the all–sample reference. Also shown are the distributions of Hamming distances for pairwise comparisons *between* sub-sampled graphs (rather than comparison to the all–sample reference graph) for Group 1 (top), Group 2 (middle), and Group 3 (bottom). The red line in each group marks the lower 5th percentile from group 3 (0.264).

In the “Group 1” tests, networks had a median Hamming distance of 0.15 compared to the reference networks (close to the ideal of 0), with a standard deviation of 0.14. In the “Group 2” tests, which subsampled the data more aggressively, the median Hamming distance was 0.28 with a standard deviation of 0.12. To visualize how network size impacts reproducibility, results of these tests were broken down by network size in Figure 4A. Looking at the normalized Hamming distance, for both Groups 1 & 2, larger networks had, on average, greater similarity to networks generated with all replicates, relative to the small networks. Larger networks also had a smaller variance in differences from the reference network. This corroborates our observation that smaller networks are more vulnerable to change, relative to larger networks.

It is reasonable to ask how this amount of similarity compares to what would be obtained from a random graph with the same sparsity and spectral properties. From the full data, we generated “spectrally–matched” graphs by applying Spielman & Srivastava’s probabilistic spectral sparsification method [8] to the same sparsification level as the all–sample reference. Repeatedly doing so enabled us to quantify the expected edge similarity between spectrally–matched random graphs (labeled “Group 3”) relative to the all–sample reference (Figure 4A). “Group 3” had a median Hamming distance of 0.32 when compared to the reference network, with a standard deviation of 0.06. Similar to Groups 1 & 2, the similarity metrics for Group 3 are visualized by network size, and again we see a larger variance among smaller networks.

We observed that the Hamming distances in Group 1 are much lower than in Group 3, indicating greater similarity than expected by chance. This suggests that random biological subsets in Group 1 retain greater structural similarity to the original network than spectrally matched random graphs (box plots in Figure 4A). This pattern was washed out in Group 2, as expected from the aggressive subsampling design. In Group 1, only one replicate per stage and cell type was removed, preserving a balanced biological representation of the full dataset and much of the underlying correlation structure. In contrast, Group 2 retained only 20% of samples, substantially reducing information. This reduction weakens correlation estimates and increases sampling variability, leading to network structures that are less consistently similar to the all–sample reference. Accordingly, the average similarity in Group 2 lies closer to that of the random graphs in Group 3 than to Group 1. However, from the box plots we observe that the larger networks in Group 2 still retain greater similarity to the original all–sample reference than the same sized networks in Group 3. This suggests that increased network size retains structural stability under subsampling and that even with only 20% of samples, meaningful biological signal is detectable.

To decipher whether the variance seen in Figure 4A is a result of the sparsification level or internal structural changes within the network, we visualized the similarity metrics as dot plots where each point is colored by the difference in sparsification level between the graph generated by the data subset versus the all–sample reference network (Figure 4B and Supplementary Figure S-1).

In Groups 1 & 2, NSE was applied to subsets of the data using the MSE-based stopping criterion, resulting in differing levels of sparsity, shown as different colors in Figure 4B. This is due to the fact that, with slightly different data, the MSE “elbow” may occur in different places. In contrast, Group 3 was sparsified such that the extent of sparsification matched that of the all–sample reference network. If we consider only graphs in Groups 1 & 2 that achieved the same level of sparsification as the reference graph (and the Group 3 graphs), the observation that the edge reproducibility is significantly higher in Groups 1 and 2 is even more pronounced (green points in Figure 4B). Notably, this is even the case for Group 2, despite the highly aggressive subsampling to just 20% of the original data. This suggests that at a given sparsification level, the detected edges remain highly reproducible even when only 20% of the samples are used. We also observe a greater range of sparsification differences in the smaller networks compared to the larger ones (Figure 4B, Groups 1 & 2), corroborating our observations above that smaller networks have a wider range of sparsification (Figure 2A). Together, this indicates that the low similarity scores observed in Groups 1 & 2 are attributable to differences in sparsification levels, whereas the higher Hamming distance scores in Group 3 are attributable to alterations in their internal network structure, given that these networks share the same sparsification level.

We repeated this analysis using the Jaccard index and spectral distance as similarity metrics (Supplementary Figure S-1). The results were similar, with one exception. In contrast to the Hamming and Jaccard metrics, the spectral distance shows larger networks have greater variance than smaller networks. This is due to the fact that the spectral distance is computed using the unnormalized graph Laplacian, and thus influenced by the network size. For an unnormalized Laplacian, the second eigenvalue *λ*_2_ (i.e., the algebraic connectivity) is bounded from above by $\frac{N}{\left(N-1\right)}min\left(d_{i}\right)$, where $d_{i}$ is the degree for node i in the graph. As a result, the maximal spectral distance between two graphs A and B (Eq. 5) is $\frac{N}{\left(N-1\right)}max\left(\underset{i\in {𝒱}_{A}}{min}\left(d_{i}^{\left(A\right)}\right),\underset{j\in {𝒱}_{B}}{min}\left(d_{j}^{\left(B\right)}\right)\right)$. This bound will generally increase as the network size increases (since the minimal degree is likely to be larger), and thus larger networks will have a wider possible range of spectral distances. This is reflected in the greater variability of spectral differences observed among larger networks.

#### 3.1.3 Utility and power of probabilistically–sparsified graphs as a spectrally–matched null model

We propose that the probabilistically–sparsified graphs (“Group 3”) can be used to form a null distribution against which graph similarities may be compared. As noted above, the Group 1 and Group 2 graphs, which are not “biologically” different from each other (or from the reference) show greater similarity (lower Hamming distance) than the Group 3 graphs. In Figure 4C–E, rather than comparing to a full–data reference, we compute the normalized Hamming distances between pairs *within* each group. With eight realizations of random–data subsets per network, each pathway (146 in total) contributes 28 similarity scores per group. Distributions of all the distances are shown in Figure 4C–E. As expected, Group 1 has greater similarity (lower Hamming distance) among pairs due to the underlying biological consistency across samples, followed by Group 2.

Following our intuition that Group 3 can be used as a null model, we next calculate what fraction of the Group 1 and Group 2 pairs have Hamming distances less than the bottom 5th percentile of Hamming distances in Group 3 — i.e., the ones for which we would (correctly) reject the null hypothesis that the graphs are no more similar than a spectrally–matched random graph. This calculation provides an estimate of the power of our proposed test. We find that 72.1% of Hamming distances in Group 1 are below the lower 5th percentile significance threshold (shown as a red line in Figure 4C–E), suggesting that we have 72.1% power to detect true similarity. In Group 2, only 14.7% of Hamming distances pass this threshold, which can be attributed to the fact that the random data subsamples in Group 2 will exclude, by chance, entire cell types and stages, and therefore should *not* be more similar than expected by chance. Indeed, we note that 18.1% of the Group 2 pairs are above the *upper* 5th percentile of the Group 3 distribution (0.44), from which we might conclude that they differ more than expected by chance.

### 3.2 Investigation of co-expression networks during development

Having assessed the similarity of network skeletons when input data varied due to random sampling, we next evaluated the differences in sparsification outcomes when the underlying biology differs. Using developing Xenopus data across six developmental stages and four cell types, we generated networks and compared the resulting differences across stages and cell types.

**Figure 5:**
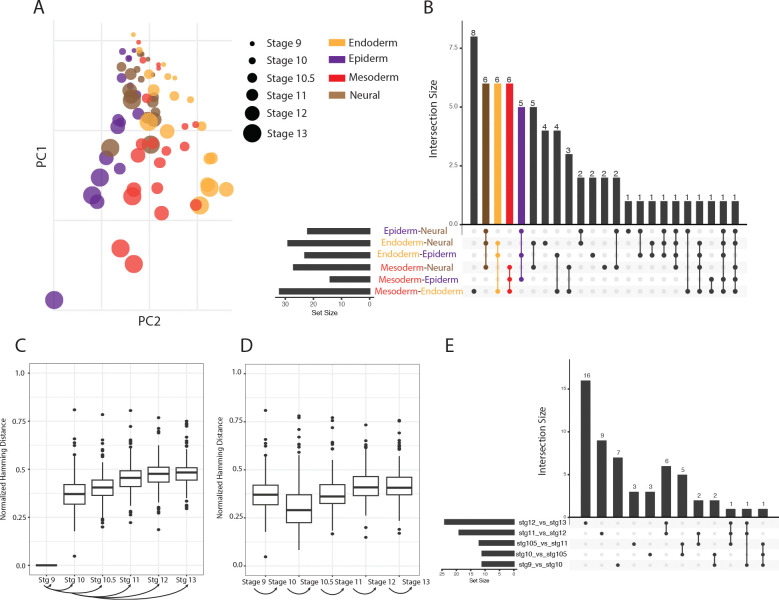
(A) PCA of Xenopus RNA-seq samples, with color indicating cell type and size indicating stage. (B-E) Generated using the normalized Hamming distance, results of other similarity metrics can be found in the supplementary materials. (B) UpSet plot indicating the number of significantly different networks between each cell-type pair. (C) Similarity scores between the reference Stage 9 network and the rest of the stages. (D) Similarity scores between each stage and its preceding stage. (E) UpSet plot indicating the number of significantly different networks between each consecutive stage.

To contextualize these comparisons, it is helpful to outline the broad developmental trends in this data. At stage 9, the cells are pluripotent and capable of giving rise to any of the four lineages, so their transcriptional profiles are largely similar across cell types. By stage 10.5, lineage differentiation becomes detectable through the expression of key transcription factors, though cells can still potentially be redirected to a different fate. By stage 13, cells have reached lineage restriction, committing to a specific fate, and each cell type exhibits a distinct transcriptional profile. The data examined here comprises four different differentiation trajectories: epidermal and neural lineages arising from an intermediary ectoderm state, and mesodermal and endodermal lineages deriving from mesendoderm. Figure 5A illustrates these dynamics.

We begin by exploring broad trends in gene expression across stages and cell types by performing principal component analysis (PCA), similar to the original analysis of the *Xenopus* transcriptomic data [34]. Figure 5A shows the first two PCs. PC1 accounts for ~39% of the variance and strongly correlates with developmental time, from early (stage 9 clustered at the top) to late development (at the bottom of the *y*-axis). PC2, which explains ~35% of the variance, articulates the final cell type, with the epiderm lineage lying farthest from the endoderm lineage, with the mesoderm lineage in-between. The neural samples remained closest to the pluripotent state, consistent with the “neural default” model [34].

We next sought to examine how the co-expression network structure changes during development. We applied NSE to build skeletons for all 146 gene sets at each cell type (across all stages) and each developmental stage (using all cell types) to explore how the network skeletons change over time and differ among cell types.

#### 3.2.1 NSE identifies networks conserved across cell types

We turned our attention to identifying which networks change the most, assessing their statistical significance compared to differences that would be expected by chance. To do so, we computed similarity scores between network skeletons generated by different cell types, “A” and “B”, and compared it to the distribution of similarity scores between the cell type “A” and the null distribution of spectrally–matched random graphs.

To measure similarity between conditions, we used three similarity metrics: normalized Hamming distance (edge-wise differences), Jaccard index (overlap in shared edges), and spectral distance (differences in global network structure). For each distribution of similarity scores, we computed a *p*-value as the proportion of random scores that were higher than the observed scores for the normalized Hamming distance and spectral distance (where larger values indicate greater differences) or lower than the observed similarity for the Jaccard Index (where smaller values indicate greater differences). The results of these tests indicate whether the observed overlap was smaller than expected by chance from a random graph, conditioned on having the same spectral characteristics.

Across the similarity metrics, we observe a consistent pattern in which each cell type shows a higher number of significantly different pathways when compared against the other cell types (Figure 5B and Supplementary Figure S-2). This suggests that each lineage maintains a distinct transcriptomic network architecture relative to the others. The recurring pattern of significantly different pathways across similarity metrics reinforces that cell identity is encoded not only in gene expression levels themselves but also in the *coordination* of expression within pathways.

Different significant pathways stand out for specific cell types. For example, the *Adherens junction pathway* (xla04520) was consistently significantly different for mesoderm relative to all other cell types, using all three similarity metrics. This likely reflects the extensive cellular rearrangements that define mesoderm development in early Xenopus. During gastrulation, mesoderm cells move inward from the embryo’s surface and spread out beneath the outer layer, forming a new internal tissue that will later give rise to muscles, bone, and other tissues [36]. Because these movements require coordinated changes in cell adhesion, adherens junctions, which are organized around the cadherin-catenin complex, play a central role. In this complex, E-cadherin mediates cell–cell adhesion and connects intracellularly to beta-catenin and alpha-catenin, linking adhesion sites to the actin cytoskeleton. Our analysis suggests that early co-regulation of these genes is essential for coordinating the collective movements that later shape mesoderm formation.

In epidermal samples, signaling pathways such as *Wnt signaling* and *ErbB signaling* emerged as consistently distinct from other cell types across all three similarity metrics, underscoring their importance in early epidermal specification and maintenance. During these stages, ectodermal cells must commit to an epidermal fate rather than a neural identity, a decision tightly regulated by Wnt activity in coordination with BMP signaling. Sustained Wnt signaling supports epidermal differentiation, whereas its inhibition shifts ectoderm toward neural fate, making this pathway particularly central in distinguishing epidermis from other lineages. Similarly, ErbB signaling contributes to epithelial proliferation, survival, and maturation. Whereas mesoderm development is marked by large-scale cellular rearrangements and neural fate is specified through BMP inhibition, epidermal development involves coordinated ectodermal expansion and differentiation processes that are modulated by growth factor–mediated signaling [37].

Additionally, epidermal samples were enriched for the *Mucin-type O-glycan biosynthesis pathway* (xla00512), a pathway responsible for the glycosylation of cell surface proteins and adhesion receptors. Mucin-type O-glycosylation modifies extracellular and membrane-bound proteins by attaching carbohydrate groups, and supports how epidermal cells adhere, communicate, and respond to growth factor signaling during epithelial sheet formation [38, 39]. These post-translational modifications reinforce epithelial stability, regulate receptor function, and help maintain the structural identity of the developing outer layer. Consistent with this interpretation, mucin glycosylation has been widely recognized as playing an essential role in eukaryotic development [40].

Endodermal samples exhibited consistent differences in the *One carbon pool by folate* pathway (xla00670) across all similarity metrics. One-carbon metabolism supports nucleotide synthesis required for DNA replication, amino acid homeostasis, epigenetic methylation reactions, and redox balance [41]. During early embryogenesis, rapid proliferation and lineage specification place high demands on these metabolic processes, and disruptions in folate metabolism impair DNA synthesis and increase the risk of developmental defects, including neural tube closure failure [42, 43]. Importantly, folate transport is mediated by Folate Receptor 1 (FOLR1), which is specifically expressed in the visceral endoderm [44], indicating that folate uptake is developmentally programmed within endodermal tissues. Together, these findings suggest that the divergence of this pathway in endodermal samples reflects its central role in coordinating proliferation and metabolic regulation during early lineage establishment.

Lastly, the *Melanogenesis pathway* (xla04916) is particularly notable, as it is significantly different in neural samples compared to other cell types across all similarity metrics. Although melanogenesis is classically associated with pigment production, melanocytes originate from the neural crest, a lineage derived from the neural plate border. In this study, neural identity was induced by Noggin treatment, which inhibits BMP signaling and shifts ectoderm toward neural fate. The enrichment of this pathway possibly reflects early activation of neural crest–associated regulatory programs embedded within the melanogenesis pathway, rather than pigment differentiation itself. The pathway may arise as significantly different in more cell type comparisons in epidermal samples than in neural samples because the later stage samples have already committed to different developmental trajectories due to BMP signaling differences.

We note a few other patterns observed across the UpSet plots: The relatively small set size for the mesoderm–epiderm comparison in all three similarity metrics suggests that, at the pathway topology level, these two lineages may be more structurally similar than other pairwise combinations (Figure 5B and Supplementary Figure S-2). It can also be observed that these lineages cluster more closely together at later stages in the PCA analysis (Figure 5A), indicating lower global transcriptomic divergence, despite each cell type being derived from different lineages. Together, both the dimensionality reduction and network-based results suggest that mesoderm and epiderm maintain comparatively similar expression and topological profiles relative to other lineages.

Secondly, the large shared intersection of significantly different pathways across nearly all cell type comparisons (excluding mesoderm–epiderm) in the Jaccard index analysis likely reflects both the underlying biological structure and properties of the similarity metric (Supplementary Figure S-2). Biologically, it suggests that many pathways undergo coordinated lineage–specific rewiring, such that each cell type adopts distinct co-expression patterns relative to the others. However, because this pattern is only seen using the Jaccard index, it may also reflect the greater sensitivity to differences in sparsity levels in this group of pathways which may cause the enrichment using this similarity metric.

A comprehensive list of all significantly different pathways across cell type comparisons and similarity metrics is provided as supplementary material.

#### 3.2.2 NSE identifies network changes over time

We next explored how the co-expression networks changed over developmental time by looking at the differences of networks from stages 10–13 compared to stage 9 networks, and identifying those that changed the most. Similarity metrics were measured between skeletons generated using stage 9 samples and the same gene set using samples from each of the other stages. As expected, Figure 5C shows a steady incline in distance between Stage 9 and other stages as cells continue to developmentally progress and cell lineages become more differentiated. Using ANOVA, we tested whether stage was a significant contributor to the variation in the stage similarity scores from stage 10 to 13; as anticipated from the boxplots, the mean change differs significantly across stages with *p* < 2e-16. Comparable trends observed using additional similarity metrics, demonstrating that this trend is observed across multiple complementary measures of network similarity (Supplementary Figure S-3).

Figure 5D and Supplementary Figure S-3 illustrates the changes in a step-wise developmental progression, that is, similarities between stages 9 and 10, between stages 10 and 10.5, stages 10.5 and 11, etc. While the common edges in stages 9 & 12 and stages 9 & 13 appear to be approximately the same in Figure 5C, this analysis reveals the median Hamming distance between stages 12 & 13 is 0.41, suggesting that their similarity to stage 9 are due to a different set of edges. Together, this global analysis of network changes among stages suggests that networks derived from distinct developmental stages are biologically different, and that those differences grow over time.

The preceding analysis considered all edges in all networks. We next turned our attention to identifying which networks change the most, assessing their statistical significance compared to differences that would be expected by chance. To do so, we computed the similarity between the stage i+1 graph and the stage i graph, and compared it to the distribution of similarity between the stage i graph and the null distribution of spectrally–matched random graphs. Similar to the cell-type analysis, the stage analysis was repeated using all similarity metrics.

We identified the networks showing the most significant structural changes (*p* < 0.05) between consecutive developmental stages and used an UpSet plot to visualize each stage transitions (Figure 5E and Supplementary Figure S-4). Notably, the later stage comparisons, particularly the transition from stage 11 to 12 and the transition from stage 12 to 13, exhibit larger set sizes relative to earlier transitions. This increase in set size suggests that a greater number of pathways show significant network differences at later developmental stages, indicating elevated variability and structural reorganization.

Although some pathways are shared across multiple comparisons, the UpSet plots reveal that the majority of significant pathways are unique to individual stage transitions or shared among only a small number of comparisons. In fact, there is only one pathway identified using the Jaccard index that is significantly different across all stage transitions (Supplementary Figure S-4A). This limited overlap suggests that changes in co-expression patterns are largely stage-specific.

#### 3.2.3 NSE identifies networks conserved across cell types and stages

Lastly, we investigated which networks have a significantly *higher* number of common edges between than expected by chance (Supplementary Figure S-5). These networks are those that differ less across conditions than would be expected by chance, and which may thus be thought of as “universal” pathways common to all conditions.

Using the normalized Hamming distance method to calculate similarity, we note the majority of significantly similar conditions arise across all pairwise comparisons for cell types (41 total) and stages (47 total). Notably, a substantial subset of pathways (18 total) overlaps between these two totals, indicating that many of the same biological programs remain conserved across both cell type and developmental stage comparisons.

Across both developmental stage and cell type comparisons, the intersecting pathways converge on a consistent set of core biological programs that form basic machinery every cell needs to survive and function across all conditions. These pathways broadly cluster into core metabolism and redox biology (supporting energy production and biosynthesis) and genetic information processing (DNA replication, transcription, RNA transport and surveillance, and protein degradation).

We identified 13 metabolism-related pathways consistently preserved across developmental stages and 17 across all cell type comparisons, including amino acid metabolism, fatty acid metabolism, and carbohydrate and energy metabolism. Similarly, 17 cellular and genetic information processing pathways were conserved across stages, and 16 across cell type comparisons, encompassing DNA replication and repair, RNA transport and surveillance, translation, protein degradation, and cell cycle regulation. The consistent presence of DNA repair and chromosomal maintenance mechanisms further suggests ongoing genome surveillance and stability across all developmental stages and cell types.

## 4 Conclusions

Here, we introduced NSE, a method for extracting the skeleton of a co-expression network via a spectral sparsification approach. NSE calculates edge importance as the product of edge weight, capturing the strength of association between two nodes, and effective resistance, capturing both the direct connection and all alternative paths between node pairs. By iteratively removing edges with low edge importance, NSE sparsifies the network while approximately maintaining the network’s spectral properties and hence its overall connectivity. The resulting network skeletons are reproducible even when constructed from random subsets of the original data.

To demonstrate NSE’s utility, we applied it to an RNA-seq dataset spanning six developmental stages and four cell types to examine how co-expression networks change over time and differ between cell types. Using a null model of spectrally–matched random graphs, we identified networks that change significantly during development, as well as those that are significantly shared between cell fates.

NSE does have some limitations. Specifically, this approach is computationally intensive, due to the need to calculate edge importance after each edge removal. While feasible for networks with fewer than a few hundred nodes, larger systems may require additional optimization or approximation techniques. Fortunately, the majority of gene sets are within the feasible range (23 of 369 human KEGG pathways; 23 of 365 mouse KEGG pathways; 34 of 157 xenopus KEGG pathways; 2 of 159 drosophila KEGG pathways).

The NSE approach for network sparsification and analysis is broadly applicable to expression data from any biological context. By preserving key spectral properties while removing low-importance edges, NSE can reveal the core structure of networks in diverse biological systems, including different tissues, conditions, or diseases. Other biological data modalities (such as ATAC-seq or metabolomics data) could also be used.

## Supplementary Material

Supplement 1

## Figures and Tables

**Table 1:** Summary of the edge importance; how a high versus low effective resistance and a high versus low edge weight affects the interpretation of that edge.

	Low edge weight *w_ij_*	High edge weight *w_ij_*
**Low effective resistance** *R_ij_*	- Weak edge - Many alternate paths - Likely remove this edge	- Strong edge - Many alternate paths - This edge is not crucial
**High effective resistance** *R_ij_*	- Weak edge - Few alternate paths - “Strength of weak ties”	- Strong edge - Few alternate paths - Crucial edge

## Data Availability

Experimental data analyzed in this study were obtained from the previously published work by Johnson et al. [34]. The experimental dataset used in this study is available in the NCBI Gene Expression Omnibus repository through GEO Series accession number GSE198598, https://www.ncbi.nlm.nih.gov/geo/query/acc.cgi?acc=GSE198598. No additional experimental data were generated as part of the present study. Code to run NSE and reproduce the figures may be obtained from https://github.com/erduvall/Network-Sparsification.

## Footnotes

* A candidate network, rather than a complete graph, can also be used if desired, reflecting prior knowledge about edges or non-edges. In either case, the starting point is a weighted, undirected graph from which we seek to remove edges.

## References

[1] Kanehisa M. and Goto S.. KEGG: kyoto encyclopedia of genes and genomes. Nucleic Acids Research, 28(1):27–30, January 2000. 10592173 10.1093/nar/28.1.27 PMC102409

[2] Milacic Marija, Beavers Deidre, Conley Patrick, Gong Chuqiao, Gillespie Marc, Griss Johannes, Haw Robin, Jassal Bijay, Matthews Lisa, May Bruce, Petryszak Robert, Ragueneau Eliot, Rothfels Karen, Sevilla Cristoffer, Shamovsky Veronica, Stephan Ralf, Tiwari Krishna, Varusai Thawfeek, Weiser Joel, Wright Adam, Wu Guanming, Stein Lincoln, Hermjakob Henning, and D’Eustachio Peter. The Reactome Pathway Knowledgebase 2024. Nucleic Acids Research, 52(D1):D672–D678, January 2024. 37941124 10.1093/nar/gkad1025 PMC10767911

[3] Savino Aurora, Provero Paolo, and Poli Valeria. Differential co-expression analyses allow the identification of critical signalling pathways altered during tumour transformation and progression. International Journal of Molecular Sciences, 21(24):9461, 2020. 33322692 10.3390/ijms21249461 PMC7764314

[4] Hsu Chia-Lang, Juan Hsueh-Fen, and Huang Hsuan-Cheng. Functional analysis and characterization of differential coexpression networks. Scientific Reports, 5(1):13295, 2015. 26282208 10.1038/srep13295 PMC4539605

[5] Katie Ovens B. Eames Frank, and Mcquillan Ian. Comparative analyses of gene co-expression networks: Implementations and applications in the study of evolution. Frontiers in Genetics, 12, 2021. 10.3389/fgene.2021.695399 PMC8414652 34484293

[6] Sipko Van Dam Urmo Võsa, Van Der Graaf Adriaan, Franke Lude, and De Magalhães João Pedro. Gene co-expression analysis for functional classification and gene–disease predictions | Briefings in Bioinformatics | Oxford Academic. 10.1093/bib/bbw139 PMC6054162 28077403

[7] Oliver Stephen. Guilt-by-association goes global. Nature, 403(6770):601–602, February 2000. Publisher: Nature Publishing Group. 10688178 10.1038/35001165

[8] Spielman Daniel A. and Srivastava Nikhil. Graph sparsification by effective resistances. In Proceedings of the fortieth annual ACM symposium on Theory of computing, STOC ‘08, pages 563–568, New York, NY, USA, May 2008. Association for Computing Machinery.

[9] Margolin Adam A., Nemenman Ilya, Basso Katia, Wiggins Chris, Stolovitzky Gustavo, Favera Riccardo Dalla, and Califano Andrea. ARACNE: An Algorithm for the Reconstruction of Gene Regulatory Networks in a Mammalian Cellular Context. BMC Bioinformatics, 7(1):S7, March 2006. 10.1186/1471-2105-7-S1-S7 PMC1810318 16723010

[10] Granovetter Mark S.. The Strength of Weak Ties1. In Leinhardt Samuel, editor, Social Networks, pages 347–367. Academic Press, January 1977.

[11] Harris Benjamin D., Crow Megan, Fischer Stephan, and Gillis Jesse. Single-cell co-expression analysis reveals that transcriptional modules are shared across cell types in the brain. Cell Systems, 12(7):748–756.e3, 2021. 34015329 10.1016/j.cels.2021.04.010 PMC8298279

[12] Braun Rosemary, Leibon Gregory, Pauls Scott, and Rockmore Daniel. Partition decoupling for multi-gene analysis of gene expression profiling data. BMC bioinformatics, 12(1):497, 2011. 22208906 10.1186/1471-2105-12-497 PMC3276603

[13] Wilk Gary and Braun Rosemary. Integrative analysis reveals disrupted pathways regulated by micrornas in cancer. Nucleic Acids Research, 46(3):1089–1101, 2018. 29294105 10.1093/nar/gkx1250 PMC5814839

[14] Gillis Jesse and Pavlidis Paul. A methodology for the analysis of differential coexpression across the human lifespan. BMC Bioinformatics, 10(1):306, 2009. 19772654 10.1186/1471-2105-10-306 PMC2761903

[15] Lemoine Gwenaëlle G., Marie-Pier Scott-Boyer Bathilde Ambroise, Périn Olivier, and Droit Arnaud. Gwena: gene co-expression networks analysis and extended modules characterization in a single bioconductor package. BMC Bioinformatics, 22(1), 2021. 10.1186/s12859-021-04179-4 PMC8152313 34034647

[16] Shah Sahil Dand Braun Rosemary. Genesurrounder: network-based identification of disease genes in expression data. BMC bioinformatics, 20(1):229, 2019. 31060502 10.1186/s12859-019-2829-y PMC6503437

[17] Tang Jianing, Kong Deguang, Cui Qiuxia, Wang Kun, Zhang Dan, Gong Yan, and Wu Gaosong. Prognostic genes of breast cancer identified by gene co-expression network analysis. Frontiers in Oncology, 8, 2018. 10.3389/fonc.2018.00374 PMC6141856 30254986

[18] WGCNA: an R package for weighted correlation network analysis | BMC Bioinformatics | Full Text. 10.1186/1471-2105-9-559 PMC2631488 19114008

[19] Bruno M Tesson Rainer Breitling, and Jansen Ritsert C. Diffcoex: a simple and sensitive method to find differentially coexpressed gene modules. BMC Bioinformatics, 11(1):497, 2010. 20925918 10.1186/1471-2105-11-497 PMC2976757

[20] Liu Bao-Hong, Yu Hui, Tu Kang, Li Chun, Li Yi-Xue, and Li Yuan-Yuan. Dcgl: an r package for identifying differentially coexpressed genes and links from gene expression microarray data. Bioinformatics, 26(20):2637–2638, 2010. 20801914 10.1093/bioinformatics/btq471 PMC2951087

[21] Amar David, Safer Hershel, and Shamir Ron. Dissection of regulatory networks that are altered in disease via differential co-expression. PLoS Computational Biology, 9(3):e1002955, 2013. 23505361 10.1371/journal.pcbi.1002955 PMC3591264

[22] Choi Younjeong and Kendziorski Christina. Statistical methods for gene set co-expression analysis. Bioinformatics, 25(21):2780–2786, 2009. 19689953 10.1093/bioinformatics/btp502 PMC2781749

[23] Chung Fan RK. *Spectral graph theory*, volume 92. American Mathematical Soc., 1997.

[24] Atay Fatihcan M., Türker Bıyıkoğlu, and Jürgen Jost. Network synchronization: Spectral versus statistical properties. *Physica D**:* Nonlinear Phenomena, 224(1–2):35–41, 2006.

[25] Batson Joshua, Spielman Daniel A., Srivastava Nikhil, and Teng Shang-Hua. Spectral sparsification of graphs: theory and algorithms. Communications of the ACM, 56(8):87–94, August 2013.

[26] Ashburner Michael, Ball Catherine A., Blake Judith A., Botstein David, Butler Heather, Michael Cherry J, Davis Allan P, Dolinski Kara, Dwight Selina S., Eppig Janan T., Harris Midori A., Hill David P., Issel-Tarver Laurie, Kasarskis Andrew, Lewis Suzanna, Matese John C., Richardson Joel E., Ringwald Martin, Rubin Gerald M, and Sherlock Gavin. Gene Ontology: tool for the unification of biology. Nature Genetics, 25(1):25–29, May 2000. Publisher: Nature Publishing Group. 10802651 10.1038/75556 PMC3037419

[27] Hou Jie, Ye Xiufen, Feng Weixing, Zhang Qiaosheng, Han Yatong, Liu Yusong, Li Yu, and Wei Yufen. Distance correlation application to gene co-expression network analysis. BMC bioinformatics, 23(1):81, 2022. 35193539 10.1186/s12859-022-04609-x PMC8862277

[28] Batson Joshua D, Spielman Daniel A, and Srivastava Nikhil. Twice-ramanujan sparsifiers. In Proceedings of the forty-first annual ACM symposium on Theory of computing, pages 255–262, 2009.

[29] Cvetković Dragoš M. . Graphs and Their Spectra. Publikacije Elektrotehničkog fakulteta. Serija Matematika i fizika, (354/356):1–50, 1971. Publisher: University of Belgrade, Serbia.

[30] Mercier Alexander, Scarpino Samuel, and Moore Cristopher. Effective resistance against pandemics: Mobility network sparsification for high-fidelity epidemic simulations. PLOS Computational Biology, 18(11), November 2022. Publisher: PLOS. 10.1371/journal.pcbi.1010650 PMC9681106 36413581

[31] Muggeo Vito M. R.. Estimating regression models with unknown break-points. Statistics in Medicine, 22(19):3055–3071, 2003. 12973787 10.1002/sim.1545

[32] Hamming Richard W. Error detecting and error correcting codes. The Bell system technical journal, 29(2):147–160, 1950.

[33] Real Raimundo and Vargas Juan M.. The probabilistic basis of jaccard’s index of similarity. Systematic Biology, 45(3):380–385, 1996.

[34] Johnson Kristin, Freedman Simon, Braun Rosemary, and LaBonne Carole. Quantitative analysis of transcriptome dynamics provides novel insights into developmental state transitions. BMC Genomics, 23(1):723, October 2022. 36273135 10.1186/s12864-022-08953-3 PMC9588240

[35] Erdős Pauland Rényi Alfréd. On the evolution of random graphs. Publ. Math. Inst. Hungar. Acad. Sci, 5:17–61, 1960.

[36] Winklbauer Rudolf. Mesodermal cell migration during xenopus gastrulation. Developmental biology, 142(1):155–168, 1990. 2227092 10.1016/0012-1606(90)90159-g

[37] Cohen Stanley. The stimulation of epidermal proliferation by a specific protein (egf). Developmental biology, 12(3):394–407, 1965. 5884352 10.1016/0012-1606(65)90005-9

[38] He Mengyuan, Zhou Xiangxiang, and Wang Xin. Glycosylation: mechanisms, biological functions and clinical implications. Signal transduction and targeted therapy, 9(1):194, 2024. 39098853 10.1038/s41392-024-01886-1 PMC11298558

[39] Zhang Jing, Dijke Peter Ten, Wuhrer Manfred, and Zhang Tao. Role of glycosylation in tgf-*β* signaling and epithelial-to-mesenchymal transition in cancer. Protein & cell, 12(2):89–106, 2021. 32583064 10.1007/s13238-020-00741-7 PMC7862465

[40] Tabak Lawrence A. The role of mucin-type o-glycans in eukaryotic development. In Seminars in cell & developmental biology, volume 21, pages 616–621. Elsevier, 2010. 20144722 10.1016/j.semcdb.2010.02.001 PMC2902666

[41] Ducker Gregory Sand Rabinowitz Joshua D. One-carbon metabolism in health and disease. Cell metabolism, 25(1):27–42, 2017. 27641100 10.1016/j.cmet.2016.08.009 PMC5353360

[42] Imbard Apolline, Benoist Jean-François, and Blom Henk J. Neural tube defects, folic acid and methylation. International journal of environmental research and public health, 10(9):4352–4389, 2013. 24048206 10.3390/ijerph10094352 PMC3799525

[43] Safi J, Joyeux L, and Chalouhi GE. Periconceptional folate deficiency and implications in neural tube defects. Journal of pregnancy, 2012(1):295083, 2012. 22900183 10.1155/2012/295083 PMC3415073

[44] Michael Salbaum J, Finnell Richard H, and Kappen Claudia. Regulation of folate receptor 1 gene expression in the visceral endoderm. Birth Defects Research Part A: Clinical and Molecular Teratology, 85(4):303–313, 2009. 19180647 10.1002/bdra.20537 PMC2731486

