# Supplementary material for: Analyzing Co-expression Networks with Network Skeleton Extraction
Source: Res Sq. 2026 Mar 25:rs.3.rs-8989183. Preprint. [Version 1] doi: 10.21203/rs.3.rs-8989183/v1 (PMC13042192; doi:10.21203/rs.3.rs-8989183/v1)
Supplement: Supplement 1 [file NIHPPrs8989183v1-supplement-1.pdf]

6 Supplementary Materials

	Endoderm	Epiderm	Mesoderm	Neural
**Stage 9**	3	6	3	3
**Stage 10**	3	6	3	3
**Stage 10.5**	3	6	3	3
**Stage 11**	3	8	5	3
**Stage 12**	3	6	3	3
**Stage 13**	3	6	3	3

Table S-1: The number of Xenopus replicates for each cell type at each stage in the experimental data set.

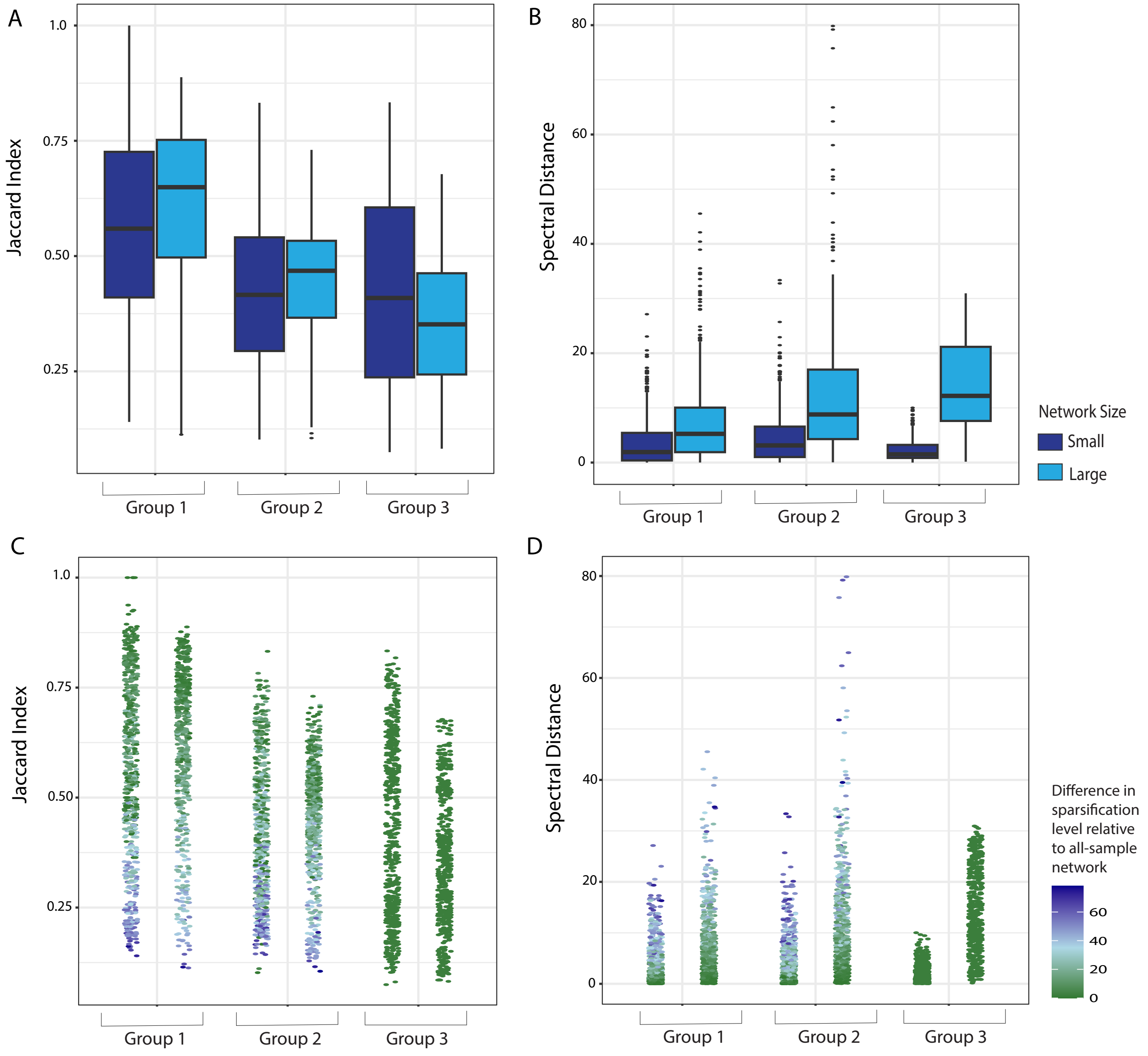

Figure S-1: Reproducibility and sensitivity to data subsampling: (A-B) Jaccard indices (=1 if exactly the same) and spectral distances (=0 if exactly the same) of three groups of networks when compared to the all–sample reference network. Plots are split by network size where small < 50 nodes ($n = 74$) and large ≥ 50 nodes (n=76)(C-D) Results in (A-B) shown as a dot plot colored by the difference in sparsification threshold between the all–sample reference network and each group.

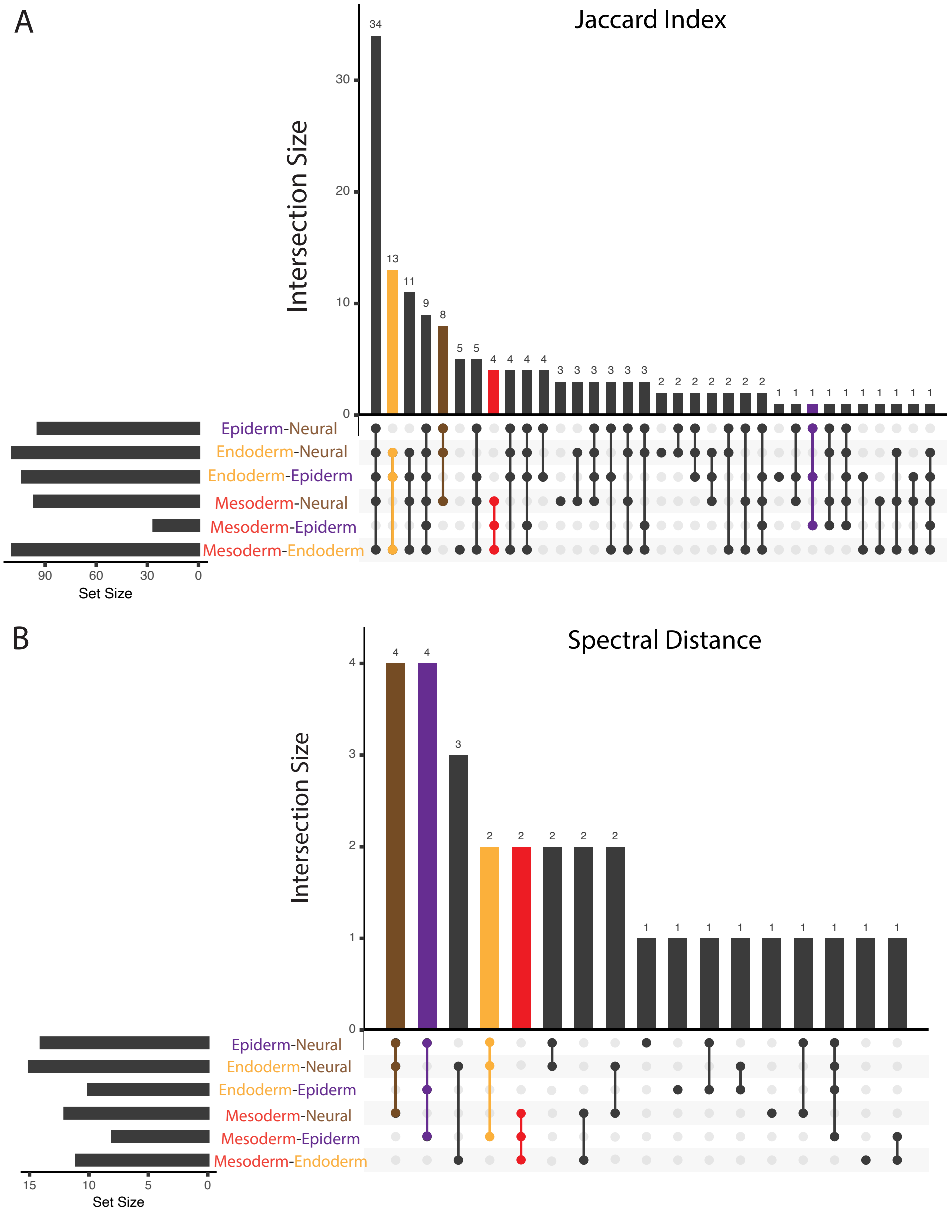

Figure S-2: Network changes between cell types: UpSet plots showing the overlap of significantly different pathways across cell type comparisons using the Jaccard index (A) and spectral distance (B).

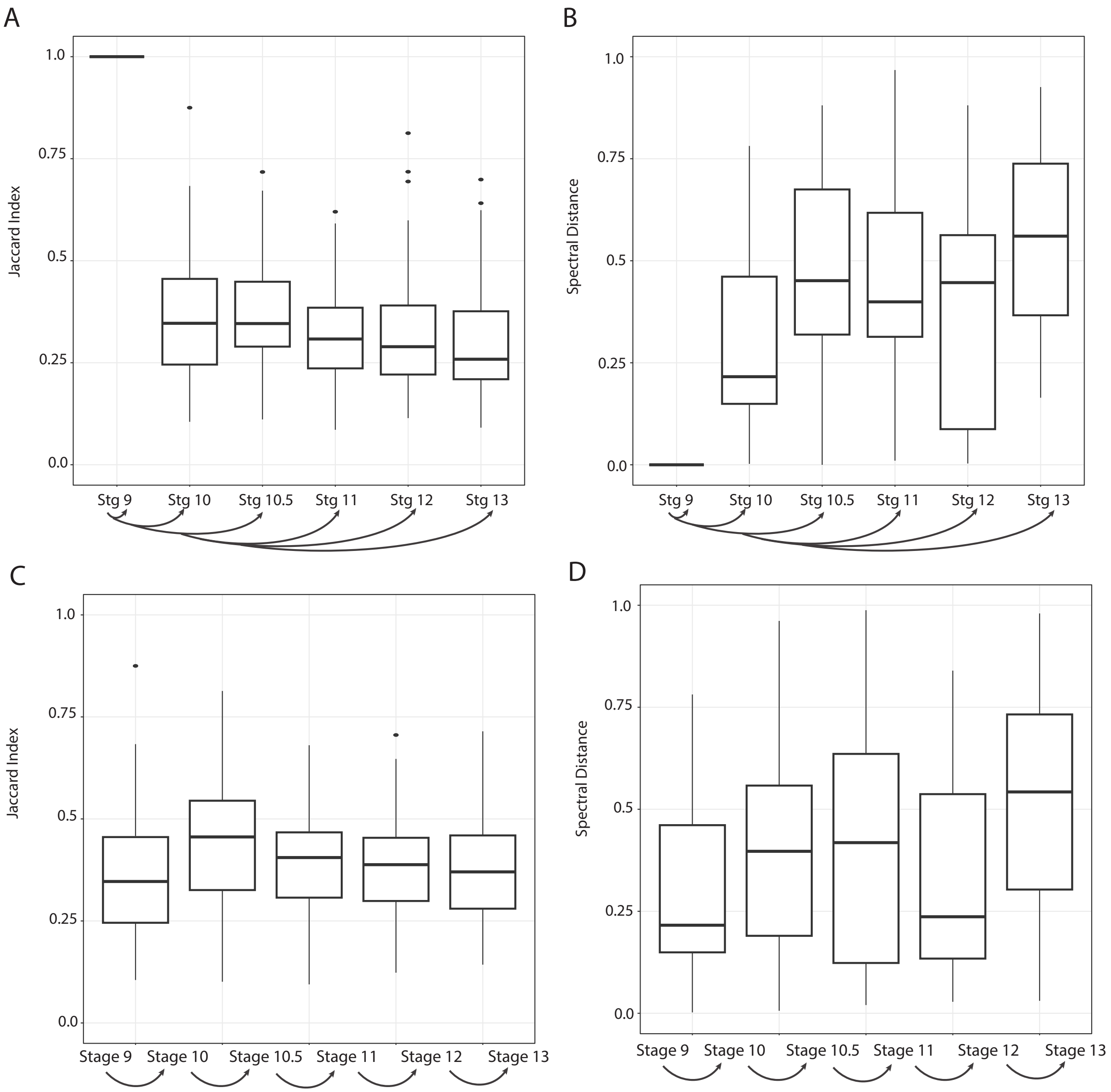

Figure S-3: Global network changes over time: Similarity scores between the reference Stage 9 network and the rest of the stages using the Jaccard index (A) and spectral distance (B). Similarity scores between each stage and its preceding stage using the Jaccard index (C) and spectral distance (D).

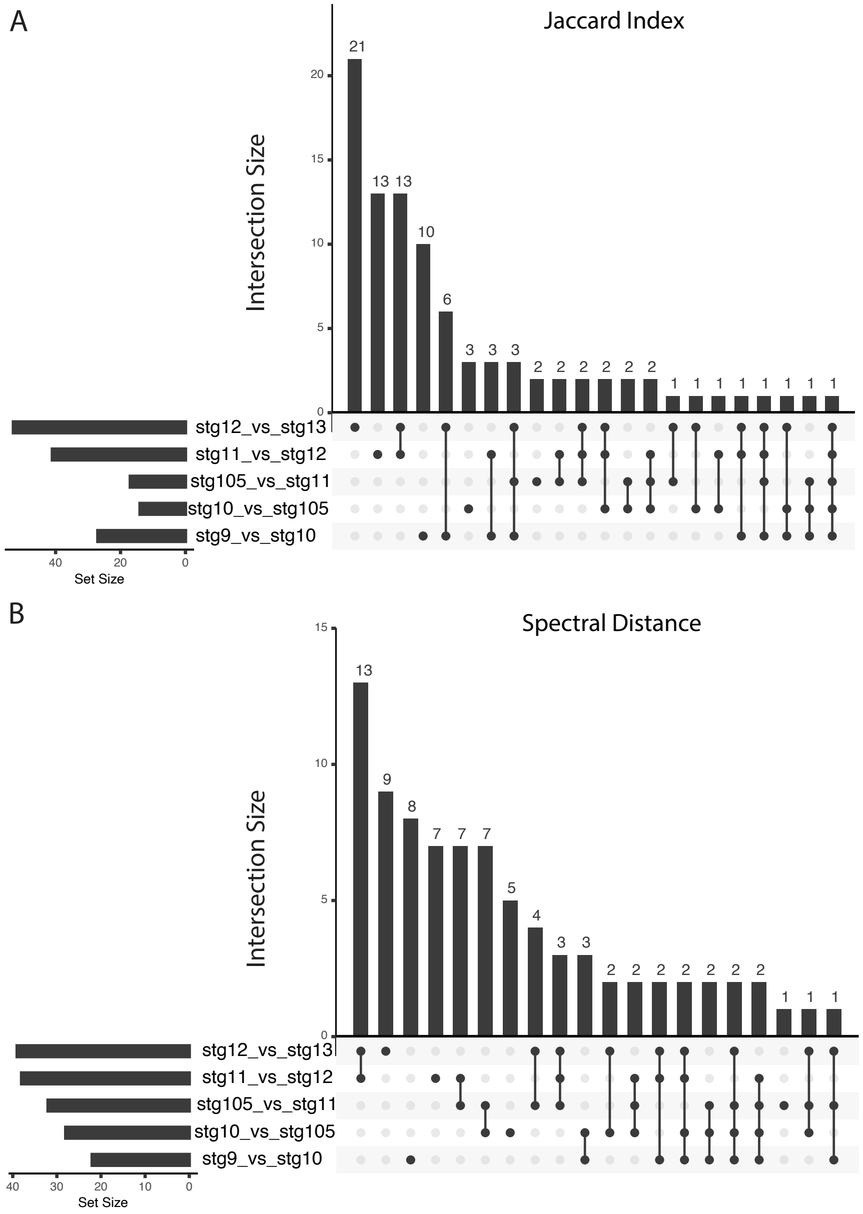

Figure S-4: Network changes over time: UpSet plots showing the overlap of significantly different pathways across stage comparisons using the Jaccard index (A) and spectral distance (B).

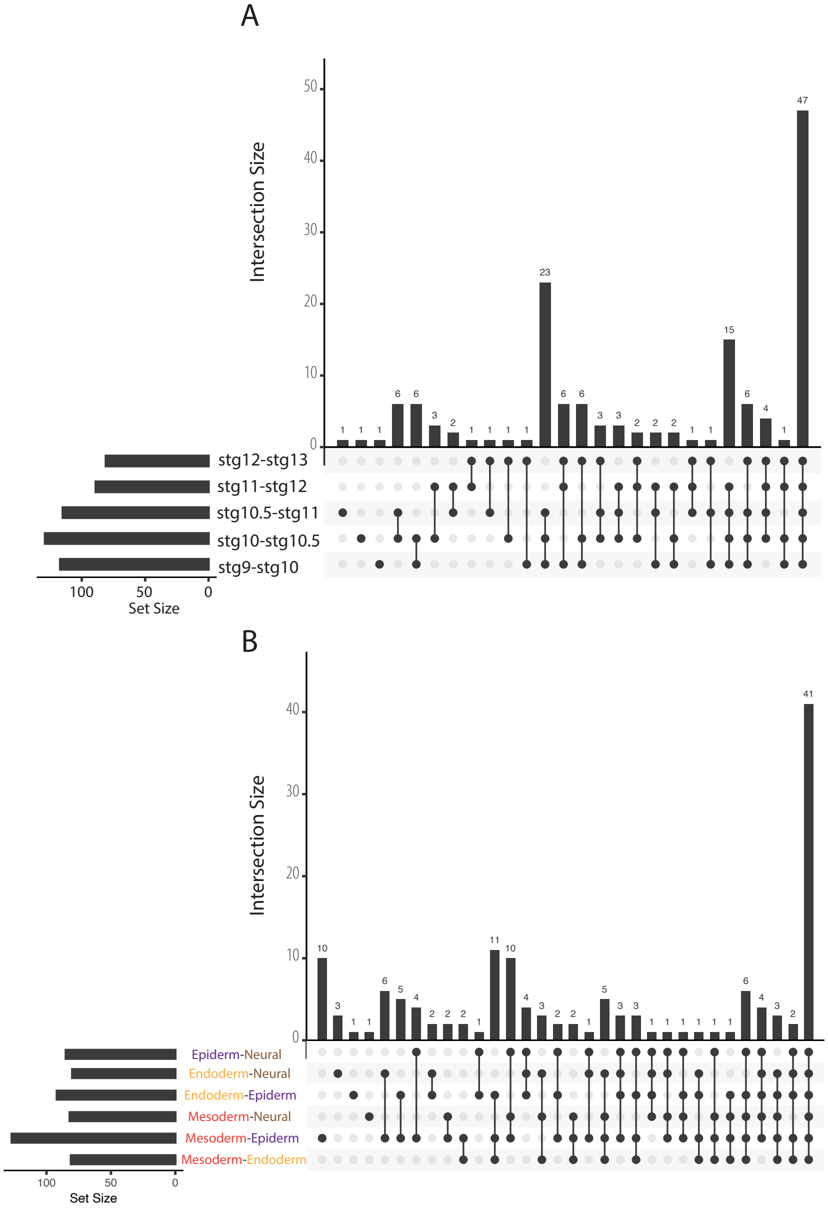

Figure S-5: Conserved networks across cell types and stages: Network similarity was assessed using normalized Hamming distance to identify pathways with significantly more shared edges than expected by chance across stages (A) and cell types (B), in which 41 conserved networks were identified across cell type pairs and 47 across stage pairs.
